# Osteolytica: An automated image analysis software package that rapidly measures cancer-induced osteolytic lesions in *in vivo* models with greater reproducibility compared to other commonly used methods^☆^

**DOI:** 10.1016/j.bone.2015.10.004

**Published:** 2016-02

**Authors:** H.R. Evans, T. Karmakharm, M.A. Lawson, R.E. Walker, W. Harris, C. Fellows, I.D. Huggins, P. Richmond, A.D. Chantry

**Affiliations:** a Sheffield Myeloma Research Team, Department of Oncology, Medical School, University of Sheffield, Beech Hill Road, Sheffield S10 2RX, UK; b Mellanby Centre for Bone Research, Medical School, University of Sheffield, Beech Hill Road, Sheffield S10 2RX, UK; c Department of Computer Science, University of Sheffield, Mappin Street, Sheffield S1 4DP, UK; d Department of Haematology, Sheffield Teaching Hospitals NHS Foundation Trust, Royal Hallamshire Hospital, Glossop Road, Sheffield S10 2JF, UK; e Insigneo Institute for *In silico* Medicine, The Pam Liversige Building, Sir Frederick Mappin Building, University of Sheffield, Mappin Street, Sheffield S1 3JD, UK

**Keywords:** Bone, Multiple myeloma, Breast cancer, Lesion, MicroCT

## Abstract

Methods currently used to analyse osteolytic lesions caused by malignancies such as multiple myeloma and metastatic breast cancer vary from basic 2-D X-ray analysis to 2-D images of micro-CT datasets analysed with non-specialised image software such as ImageJ. However, these methods have significant limitations. They do not capture 3-D data, they are time-consuming and they often suffer from inter-user variability. We therefore sought to develop a rapid and reproducible method to analyse 3-D osteolytic lesions in mice with cancer-induced bone disease. To this end, we have developed Osteolytica, an image analysis software method featuring an easy to use, step-by-step interface to measure lytic bone lesions. Osteolytica utilises novel graphics card acceleration (parallel computing) and 3-D rendering to provide rapid reconstruction and analysis of osteolytic lesions. To evaluate the use of Osteolytica we analysed tibial micro-CT datasets from murine models of cancer-induced bone disease and compared the results to those obtained using a standard ImageJ analysis method. Firstly, to assess inter-user variability we deployed four independent researchers to analyse tibial datasets from the U266-NSG murine model of myeloma. Using ImageJ, inter-user variability between the bones was substantial (± 19.6%), in contrast to using Osteolytica, which demonstrated minimal variability (± 0.5%). Secondly, tibial datasets from U266-bearing NSG mice or BALB/c mice injected with the metastatic breast cancer cell line 4T1 were compared to tibial datasets from aged and sex-matched non-tumour control mice. Analyses by both Osteolytica and ImageJ showed significant increases in bone lesion area in tumour-bearing mice compared to control mice. These results confirm that Osteolytica performs as well as the current 2-D ImageJ osteolytic lesion analysis method. However, Osteolytica is advantageous in that it analyses over the entirety of the bone volume (as opposed to selected 2-D images), it is a more rapid method and it has less user variability.

## Highlights

• We designed Osteolytica software to automatically detect and measure bone lesions.
• Software is as good as the current best method of lesion analysis.
• Software results in less inter-user variability than the current best method.
• Osteolytica has the potential to be used in clinical care.

## 1 Introduction

A classical feature of malignancies such as multiple myeloma (MM) and metastatic breast cancer (MBC) is a destructive bone disease featuring the formation of focal osteolytic lesions [1], [2]. In both cases, formation is driven by increased osteoclastic bone resorption and reduced osteoblastic bone formation, specifically mediated by osteoclast activating factors [3], [4] and osteoblast inhibitory factors [5], [6] secreted by malignant cancer cells. Osteolytic lesions are considered a cardinal sign of MM [7] and MBC [8], and assessment of them is of substantial clinical importance.

A number of *in vivo* murine models of MM and MBC are in use worldwide as a platform to study the biology of these diseases and the efficacy of new therapeutic agents against tumour growth and associated bone disease. For MM, these include the 5TMM murine myeloma syngeneic series (5T2MM, 5T33MM and 5TGM1) [9], [10], [11] and various immune deficient xenograft models using NOD/SCID [12], [13], SCID-hu [14], [15], [16], SCID-beige [17], SCID-Rab [18], [19], [20] and more recently NOD/SCID-γ mice [21], [22], [23]. There are also transgenic models of MM, including mice that are genetically altered to over-express *MYC* [24], *Myc*/*B cell extra-large* (*BCl-XL*) [25] and *X-box binding protein 1* (*XBP-1*) [26]. For MBC, these include the 4T1 murine breast cancer syngeneic model [27] and the MDA-MB-231 xenograft model [28]. As with the human diseases, all of these models demonstrate the formation of osteolytic lesions as tumour load increases. These lesions can vary considerably from a relatively low number of large lesions to a higher number of smaller lesions. The analysis of osteolytic lesions, usually recorded as a number or an area, is used as a marker of disease progression and to assess the effects of therapies designed to reduce bone disease. However, given the complex 3-D structure of bone, capturing the full effects of therapeutic agents on bone disease is deceptively difficult. Methods used to analyse osteolytic lesions have included crude counts conducted on poor quality 2-D plain radiographs [29] through to more technologically advanced methods that utilise imaging software, such as ImageJ (v. 1.47 t, NIH, USA) applied in various ways to first 2-D X-rays [30] and later 3-D micro-CT reconstructions [31]. These methods are time-consuming, inaccurate, and suffer from inter-user variability that leads to poor reproducibility of results and possible user bias.

To tackle these problems we have designed Osteolytica, a software package for the analysis of cancer-induced osteolytic lesions featuring an easy to use, step-by-step interface. It utilises novel graphics card acceleration (parallel computing) of localised surface reconstruction techniques and 3-D volume rendering to provide rapid reconstruction and analysis of osteolytic lesions. In this report, we firstly assessed the inter-user variability of results obtained using Osteolytica compared to those obtained with ImageJ analysis. Secondly, we compared the results, using both methods, from a xenograft model of myeloma study and a murine metastatic breast cancer study.

## 2 Methods

### 2.1 Cell lines

U266 cells (derived from the peripheral blood of a 53-year-old man with MM) were purchased from LGC Standards (UK). 4T1 murine breast cancer cells (originally derived from a spontaneous mammary tumour in a BALB/c mouse [27]) were kindly donated by Prof. Janine Erler (Biotech Research & Innovation Centre, University of Copenhagen, Copenhagen, Denmark). Cell lines were genetically profiled by DSMZ and ATCC using short tandem repeat analysis to confirm their identity. U266 cells were cultured in RPMI 1640 medium containing 10% foetal calf serum (FCS), 100 U/ml penicillin and 100 μg/ml streptomycin. 4T1 cells were cultured in DMEM medium containing 10% FCS, 100 U/ml penicillin and 100 μg/ml streptomycin. For both cell lines, all reagents were from Gibco, Paisley, UK. Cells were cultured at 37 °C in 5% CO_2_ until required.

### 2.2 Mice

NSG (NOD.Cg-Prkdcscid Il2rgtm1Wjl/SzJ) mice and BALB/c mice were purchased from Charles River (Margate, UK).

### 2.3 Ethics statement

All procedures involving animals were approved by the Home Office (PPL 40/3462) and the University of Sheffield's Animal Ethics Committee.

### 2.4 *In vivo* studies

#### 2.4.1 U266-NSG myeloma model

8–9 week-old male NSG mice were divided into a non-tumour control group (n = 4) and a tumour group (n = 4). Mice in the control group were injected intravenously (i.v.) with 100 μl PBS *via* the tail vein. Mice in the tumour group were injected i.v. with 1 × 10^6^ U266 cells. At the first signs of morbidity, at 8 weeks post-tumour cell injection, all mice were sacrificed.

#### 2.4.2 4T1 breast cancer model

10 week-old female BALB/c mice were divided into a non-tumour control group and a tumour group (n = 6/group). Mice in the control group were injected with 100 μl PBS into the mammary fat pad. Mice in the tumour group were injected with 1 × 10^5^ 4T1 cells into the mammary fat pad. All mice were sacrificed at 14 days post-tumour cell injection.

For all studies, the right tibiae were dissected free of soft tissue and fixed in 10% neutral buffered formalin.

### 2.5 Micro-CT analysis

Tibiae were scanned on a Skyscan micro-CT scanner (1172a, Bruker, Belgium) at 50 kV and 200 μA using a 0.5 mm aluminium filter and a detection pixel size of 4.3 μm. Images were captured every 0.7° through an 180° rotation with a 2 × averaging of each bone. Scanned images were reconstructed using Skyscan NRecon software (v. 1.6.9, Bruker, Belgium) and datasets were resized using Skyscan CTAn (v. 1.14.4, Bruker, Belgium).

### 2.6 ImageJ: 2-D analysis of osteolytic lesions

Trabecular bone was firstly removed from the datasets, leaving only the cortical bone shell, using Skyscan CTAn. Datasets were volumetrically rendered using Drishti (v. 1.0, ANU Vizlab, Australia), and then imaged. Tibiae were taken to be roughly triangular in cross-sectional profile, and so images were taken of these three different sides (the concave face, the face adjacent to the back of the fibular and the flat face), each time with the bone clipped in half so that any lesions showed through to the background colour behind. Images were then analysed using ImageJ (v. 1.47t, NIH, USA). Each image was binarised and thresholded so that bone lesions appeared as areas of red. The surface areas of these red regions were measured and the total surface area of all the lesions as a proportion of the bone area was then calculated for each bone.

### 2.7 Osteolytica: 3-D analysis of osteolytic lesions

A volumetric dataset from a micro-CT scanner was imported into Osteolytica and the maximum diameter for a single bone lesion specified. Maximum diameter was specified manually at 900 μm. The software first expanded the sample bone volume until there were no holes on the outer surface. A contraction process was then performed from the outside surface of the expanded volume, which continued until the highest overlapping ratio between the contracted surface and the original sampled bone volume was found. This determined the optimal reconstruction of the sampled bone volume. The addition of the contracted volume to the original sampled bone volume formed the final bone reconstruction producing osteolytic lesion counts and area measurements.

### 2.8 Statistical analysis

All data were assumed to be non-parametric and analysed using a Mann–Whitney test. Significance is indicated where p < 0.05 and all data are expressed as mean ± SD.

## 3 Results

### 3.1 Osteolytica is a user-friendly interface that rapidly measures the number and area of osteolytic lesions

**Fig. 1:**
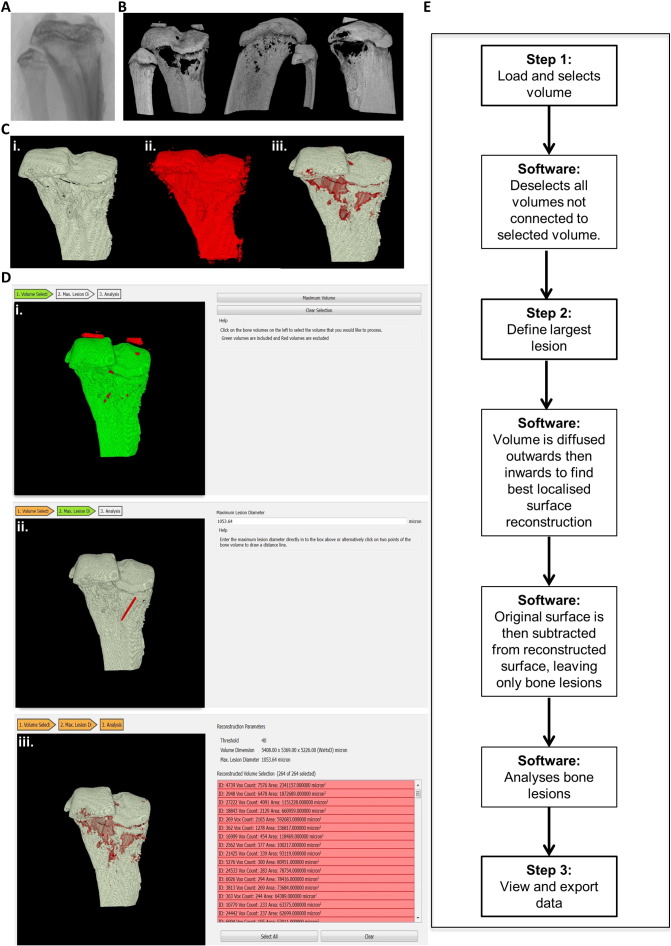
Osteolytica is a user-friendly interface that rapidly measures the number and area of osteolytic lesions. (A) Plain radiograph of a mouse tibia used as the original template for manual lesion counts. (B) Micro-CT 2-D reconstruction of a mouse tibiae showing the 3 aspects used as templates for 2-D ImageJ analysis to assess lesion area and number. (C) The 3 stages of Osteolytica, showing (i.) volume selection, (ii.) volume expansion and (iii.) detection and measurement of lesions. (D) Representative screenshots of the 3 steps of the Osteolytica software showing (i.) volume selection, where the user loads the dataset and selects the volume of interest, and the software removes any unattached floating volumes (ii.) maximum lesion diameter specification, where the user either manually enters the diameter or draws it on the screen and (iii.) lesion analysis results, where the software lists the area of every lesion measured, as well as total lesion area and total lesion proportion of the volume. Lesions can be deselected at this stage if required. (E) Flow diagram showing the 3 steps of Osteolytica and intermediate computer processes.

Osteolytica was designed to be easier, faster and less biased than previous lesion analysis methods such as the manual counting of lesions evident on an X-ray (Fig. 1A) or ImageJ 2-D analysis (Fig. 1B). Osteolytica was developed solely to count the number and area of osteolytic lesions of a 3-D bone volume, thereby quantifying the full extent of any cancer-induced bone disease (Fig. 1C). It was not designed to measure standard micro-CT parameters, such as trabecular volume and spacing, and should therefore be seen as an addition to standard micro-CT software and not a replacement. The datasets used in this publication were obtained using a Skyscan micro-CT scanner, but datasets from other micro-CT scanner, such as Scanco, and clinical CT scanners would also be compatible.

The software features an easy to use, step-by-step interface preceded by a file selection interface, which allows users to input a volumetric sample and apply a binary threshold. The first step of the analysis process is for a user to provide a volume selection. The user loads the desired dataset, specifies the pixel resolution of that dataset and clicks on it, and the software then deselects any disconnected volumetric areas from the main area of interest, such as pieces of floating patella (Fig. 1Di.). The second stage involves specifying a maximum lesion diameter. The user can manually type in a diameter, or they can click across the lesion on-screen and the software will automatically input that diameter (Fig. 1Dii.). The final stage provides analysis results (output) of the sample volume by showing the lesion analysis measurements (Fig. 1Diii.). Every lesion found is listed individually in order of size, and below the total lesion area as a proportion of the bone is displayed. During this final stage individual osteolytic lesions can be removed through 3-D picking or *via* de-selection from a list of lesions sorted by lesion size, for example, to remove naturally occurring holes such as those for blood vessels. In total, the user completes 3 steps and the software carries out 4 separate processes (Fig. 1E). Other than dataset resizing Osteolytica requires no other dataset preparation, unlike the 2-D ImageJ method that calls for lengthy volumetric rendering and image capture of the dataset prior to lesion analysis. In total, using the 2-D ImageJ method requires 8 separate processes (dataset resizing, trabecular hollowing, volume rendering, model slicing, slice imaging, image cropping, image size standardising, ImageJ analysis) and takes approximately 20 min per dataset. Comparatively, using Osteolytica only requires 4 (dataset resizing, Osteolytica dataset loading, Osteolytica maximum lesion size selecting, Osteolytica lesion analysis) and takes approximately 5 min per dataset. In our experience, using Osteolytica is therefore four times faster than using the 2-D ImageJ method. The software can be found at the following link: http://www.osteolytica.com and downloaded for a small fee, the proceeds of which are used to cover developmental costs.

The method of calculating lesions within Osteolytica uses a process of reconstructing the surface of a volumetric sample (filling lesions) and then subtracting the original surface from the reconstructed one (Fig. 1C). Reconstruction is implemented through a novel two-stage process of volumetric diffusion [32]. Within the first stage of this process the volumetric surface is diffused outwards (according to the user specified maximum lesion diameter) (Fig. 1Ci. and ii.). This expanded volume is then diffused inwards by a variable amount over the surface (Fig. 1Ciii.). The variable inwards diffusion is optimised to find the highest overlapping ratio between the contracted surface and the original sampled bone volume over a fixed local area (based on the maximum lesion diameter). This has been found to provide the best localised surface reconstruction. As the volumetric diffusion process is computationally expensive this aspect of the software has been accelerated through the use of parallel processing using the graphics processing unit [33].

### 3.2 Osteolytica has much lower inter-user variability and therefore much better reproducibility compared to ImageJ

**Fig. 2:**
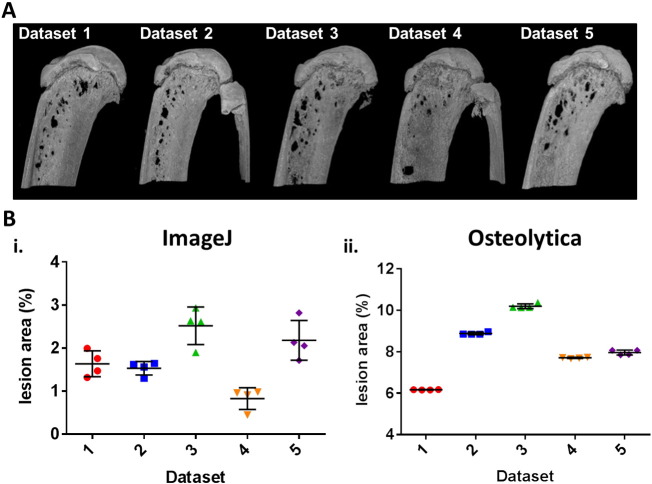
Osteolytica reduces inter-user variability compared to a 2-D ImageJ analysis method. (A) Representative micro-CT full-face images of tibia (n = 5), from mice injected with 1 × 10^6^ U266 cells at the end stage of disease, used to assess inter-user variance. (B) Bone lesion area showing inter-user variance when using the 2-D ImageJ method (19.6%) (i.) and when using Osteolytica (0.53%) (ii.).

To assess the inter-user variability of Osteolytica compared to a 2-D ImageJ analysis method, 4 independent researchers analysed the same 5 datasets of micro-CT bone images using both methods. For both methods, analysis of bone destruction was measured as total lesion area as a proportion of the region of interest. For the 2-D ImageJ method, this meant percentage of the bone area imaged. For the 3-D Osteolytica method, this meant percentage of the total bone area. This parameter was chosen as it was felt that total lesion area gave a truer reflection of the extent of bone destruction as opposed to simple numbers of osteolytic lesions. These datasets were tibiae from NSG mice injected with U266 myeloma cells and sacrificed at the end stage of disease. Osteolytic lesions could be visualised in all of the bones (Fig. 2A). Using the ImageJ analysis method (Fig. 2Bi.), when collated inter-user variability for dataset 1 was 18.4%, for dataset 2 was 10.3%, for dataset 3 was 17.4%, for dataset 4 was 30.6%, and for dataset 5 was 21.2%. Thus, the average inter-user variability was found to be 19.6%. In contrast, analysis using Osteolytica (Fig. 2Bii.) resulted in 0.1% variability for dataset 1, 0.76% for dataset 2, 0.13% for dataset 3, 0.89% for dataset 4 and 0.74% for dataset 5. Average inter-user variability using Osteolytica was 0.53%, which was substantially lower than when using the 2-D ImageJ method.

### 3.3 Osteolytica and ImageJ are both able to detect significant differences in myeloma-induced osteolytic lesions

**Fig. 3:**
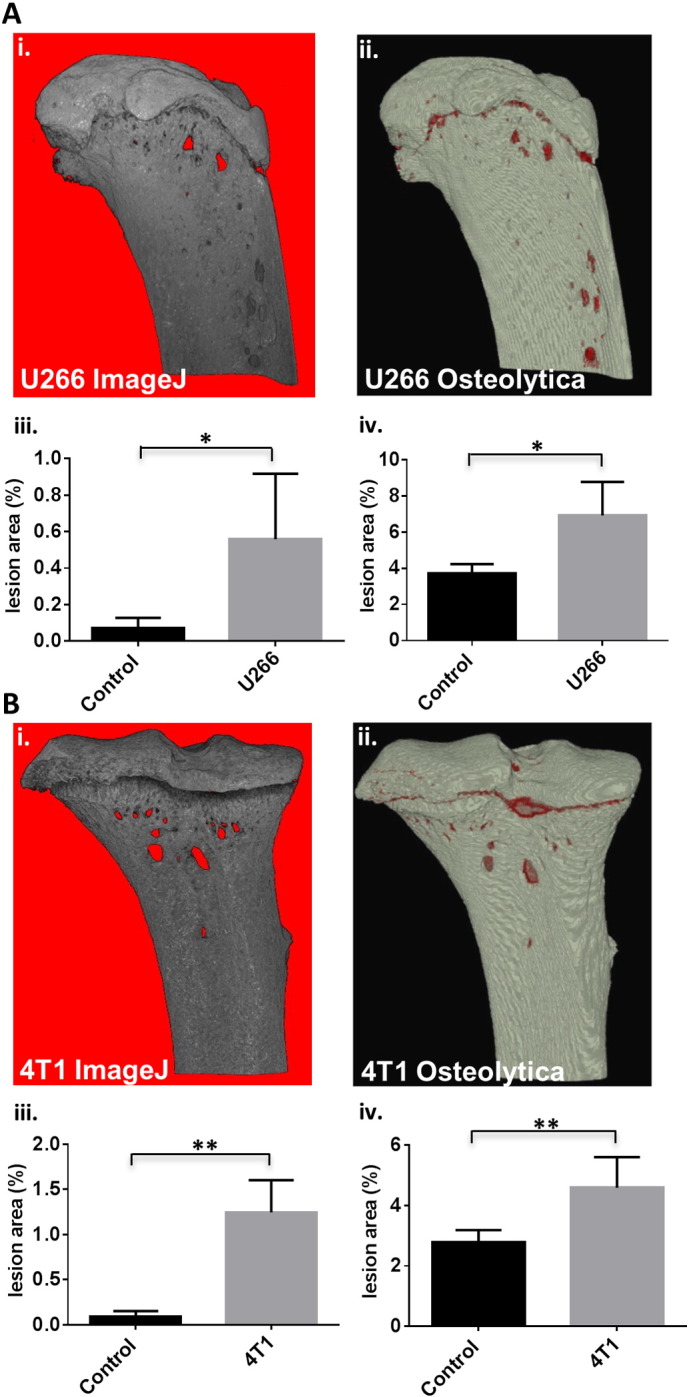
Osteolytica is able to detect significant differences in osteolytic lesion area in micro-CT scans from murine models of cancer-induced bone disease. (A) Representative images of tibiae from mice injected with 100 μl PBS (Naïve, n = 4) (i.) or 1 × 10^6^ U266 cells (n = 4) (ii.). Bone lesion area when using the 2-D ImageJ method (0.56 ± 0.36% *vs* 0.07 ± 0.058%) (iii.) and when using Osteolytica (6.9 ± 1.9% *vs* 3.7 ± 0.5%) (iv.). (B) Representative images of tibiae from mice injected with 100 μl PBS (Naïve, n = 6) (i.) or 1 × 10^4^ 4T1 cells (n = 6) (ii.). Bone lesion area when using the ImageJ method (1.25 ± 0.36% *vs* 0.089 ± 0.064%) (iii.) and when using Osteolytica (4.6 ± 1.0% *vs* 2.8 ± 0.4%) (iv.). Data are expressed as mean ± SD and significance from the control group is indicated, where *p < 0.05 and **p < 0.01.

To compare Osteolytica lesion analysis to ImageJ analysis, datasets from NSG mice injected with the U266 cells were assessed and compared to non-tumour controls (Fig. 3A). For both methods, analysis of bone destruction was measured as total lesion area as a proportion of the region of interest. The tumour group had a significantly larger total lesion percentage compared to the non-tumour group when using both the ImageJ 2-D method (Fig. 3Ai.) and Osteolytica (Fig. 3Aii.). Using ImageJ (Fig. 3Aiii.), there was a significant difference of p < 0.05 between the tumour group and the naïve group (0.56 ± 0.36% *vs* 0.070 ± 0.058%). Similarly using Osteolytica (Fig. 3Aiv.), there was a significant difference of p < 0.05 for the tumour group *versus* the non-tumour group (6.9 ± 1.9% *vs* 3.7 ± 0.5%).

### 3.4 Osteolytica can be used to assess osteolytic lesions in a breast cancer murine model

Next, we assessed the use of Osteolytica compared to the 2-D ImageJ method in a different murine model of cancer-induced bone disease. Tibial micro-CT datasets from BALB/c mice injected with 4T1 breast cancer cells were assessed compared to non-tumour mice (Fig. 3B). For both methods, analysis of bone destruction was measured as total lesion area as a proportion of the region of interest. The tumour group had a significantly larger total lesion percentage compared to the non-tumour group when using both analysis methods (Fig. 3B). Using ImageJ (Fig. 3Biii.), there was a significant difference of p < 0.01 for the tumour group *versus* the non-tumour group (1.25 ± 0.36% *vs* 0.089 ± 0.064%). Similarly using Osteolytica (Fig. 3Biv.), there was a significant difference of p < 0.01 between the tumour group and the non-tumour group (4.6 ± 1.0% *vs* 2.8 ± 0.4%).

## 4 Discussion

Cancer-induced bone destruction leading to severe pain, immobility and disfigurement is one of the most devastating aspects of the patient's experience of cancer [1]. New treatments to prevent osteolytic bone disease and to eliminate cancer from the bone marrow microenvironment are urgently needed. Current therapy relies almost exclusively on bisphosphonates, which have been proved to reduce the skeletal complications of malignancy [34], [35], [36], [37], [38] but do not lead to repair of existing lesions.

To assess new and existing therapies in the treatment of cancer-induced bone disease both immune-competent syngeneic and human xenograft *in vivo* murine models are used. Some of these models exhibit severe osteolytic phenotypes including distinct lesions. These models have provided important evidence concerning the efficacy of both anti-resorptive agents such as the bisphosphonate, zoledronic acid [29], [39], proteasome inhibitors such as bortezomib [40], osteoprotegrin mimetics [19] and anabolic agents such as anti-Dkk-1 [30] (*Novartis*), and the decoy activin receptor, RAP-011 (*Acceleron*) [41]. However, in order to robustly assess these new reagents it is imperative that osteolytic lesions are measured accurately and without bias. It has become apparent that methods used to analyse the number and area of osteolytic lesions are sub-optimal, especially with respect to inter-user variability, reproducibility and consequent accuracy.

Here we have demonstrated a new software method, known as Osteolytica, and compared it to an existing method using 2-D ImageJ analysis, to measure osteolytic lesions from murine micro-CT datasets. Osteolytica features a user-friendly, step-by-step digital interface, which is easily mastered and quick to use. It allows complete 360° analysis of the bone's surface, unlike the 2-D ImageJ method that only allows for partial bone analysis, the results of which could skew analysis. This is illustrated in the murine study examining the inter-user variability between the two analysis methods (Fig. 2). In this study, the full-face images of the tibiae (Fig. 2A) appear to show that Dataset 4 has the least osteolytic disease, and this is reflected in the ImageJ method results. It is only when using Osteolytica, and thus considering the entirety of the bone, that it becomes apparent that Dataset 1 is actually the least osteolytic.

The software details and tags each individual lesion, as well as calculating total bone destruction present, and this can be displayed both as a surface area and as a proportion of the total bone area. This tagging feature does allow the user to remove naturally occurring holes such as blood vessels if they wish — for example, most mice tibiae have a blood vessel on the curved surface opposite to the back of the fibula. Removal of blood vessels, therefore, does require the user to have a certain level of expertise. It is inadvisable in many cases, as cancer-induced bone disease can widen already naturally occurring holes. It can also be difficult to pin-point the bone vessel hole in a heavily diseased bone that it full of lesions. Finally, as Osteolytica is fully automated there is much less scope for user bias from analysis of data. We have shown that repeated analysis of the same dataset by different users yields the same result, whereas results differ markedly when using the existing method using 2-D ImageJ analysis.

Comparing the results obtained from the two murine studies, it appears that the 2-D ImageJ method actually produces better results than when using Osteolytica. However, this would be a misinterpretation, because obtaining a good result from a technique does not necessarily mean that that technique is correct. As we have mentioned above, using the ImageJ method only offers a partial analysis of the bone using three 2-D images. 2-D images of a 3-D structure can flatten and distort its features, particularly curved surfaces, leading to inaccurate measurements. Finally, the amount of volume manipulation and bone imaging required increases the chance for user error and decreases the likelihood of reproducibility. Therefore, we would argue that though the results gained by using Osteolytica may not look as impressive as those produced from other methods on the surface, they are almost certainly more accurate. Currently, there is no established way of knowing the true value of a bone's osteolytic disease. Micro-CT allows accurate and complete visualisation of bone lesions but its supporting computer software offers no method of capturing this information, hence the reason why we designed Osteolytica. Therefore, although it is difficult for us to test the accuracy of Osteolytica we are confident that for the reasons discussed, Osteolytica gives the most accurate analysis of bone destruction currently possible.

Presently, there is only one published methodology for the analysis of bone destruction in 3-D, which uses a novel algorithm to ‘unwrap’ the cortical bone and produce a flat, 2-D image that can be analysed to measure lesion number and area. This method of analysis is a marked improvement from the 2-D ImageJ method, the problems of which we have detailed above. However, this unwrapping method still has its limitations, and we believe that Osteolytica demonstrates an improvement on this 3-D method. The unwrapping method is only applied to the cylindrical portion of the bone below the growthplate, most likely because the unwrapping algorithm cannot deal with the more complex, curved surface of the growthplate and the epiphysis above it. Thus, a large portion of the bone is excluded, and this is made more problematic by the fact that many models of osteolytic disease develop lesions primarily in that growthplate region, due to the cortical wall being much thinner and more porous. A large proportion of disease would therefore avoid detection. Osteolytica can cope with the complexities of the shape of the bone in its entirety through the use of its two-stage volumetric diffusion method, so no region of the bone is ignored and no data is lost.

Osteolytica is, however, designed primarily to analyse the cortical bone of long bones. It can analyse lesions in flat bones, such as the calvaria, but it has not been optimised for complex structures that contain very acute angles, as the software will take those angles to be lesions. It would therefore not be appropriate to use Osteolytica to analyse lesions in vertebrae or in trabecular bone. Very large datasets would also require some resizing and pixel smoothing, which may lead to the loss of very small holes.

The successful deployment of Osteolytica in the detection and measurement of osteolytic lesions in *in vivo* models of cancer-induced bone destruction paves the way for its deployment as an additional diagnostic modality in patients with cancer-induced bone disease. The detection of cancer induced bone disease in patients remains a crucial issue. In the case of myeloma, detection of bone disease is a critical aspect of diagnosis, being a key determinant of whether to commence chemotherapy [42]. The optimum radiological method of assessing bone destruction in myeloma is currently the subject of substantial international debate [43]. Plain radiography remains the screening modality of choice in the detection of osteolytic lesions, given that it is universally available and inexpensive [44]. CT, magnetic resonance imaging (MRI) and positron emission tomography–computerised tomography (PET/CT) are frequently used to clarify ambiguous lesions on X-ray or anatomical sites of unexplained pain or suspected malignant infiltration [43].

As shown, Osteolytica is able to detect and analyse bone lesions from murine micro-CT datasets. We anticipate that the application of Osteolytica to human CT datasets would be entirely feasible once conditions are optimised. Datasets would need to be high-definition scans, *i.e.* of sufficiently small pixel resolution to allow identification of lesions, and to contain a sufficient number of image slices in order to produce a smoothly defined 3-D model. Generally speaking, there should be a 4:1 ratio between the object of interest and the minimum pixel resolution, so to detect a lesion of 20 μm diameter the pixel resolution should be at least 5 μm. Because of the resultant file size from these optimisations, the datasets would probably require a degree of resizing and pixel smoothing. Once these conditions were met, however, Osteolytica would provide a novel and powerful method of detecting and quantifying cancer-induced bone destruction. Furthermore, serial measurements using Osteolytica would enable the detection of new or progressive disease, and could also monitor response to bone targeted treatments including repair of existing lesions driven by the application of bone anabolic agents. We are currently seeking ethical approval to apply Osteolytica to human CT datasets.

In summary, Osteolytica software is quick and easy to use. It performs as well as the current 2-D ImageJ method of analysing osteolytic lesions, but also substantially reduces inter-user variability. Osteolytica, therefore, has the potential to become the international standard method of analysis in the *in vivo* research context. In addition, Osteolytica has a potentially important clinical role as a better detection method of bone disease. This has important implications for treatment decisions, including when to commence chemotherapy and when to call for orthopaedic intervention.

## Conflict of interest

None.
